# Characterization and Genomic Analysis of a New Phage Infecting *Helicobacter pylori*

**DOI:** 10.3390/ijms23147885

**Published:** 2022-07-17

**Authors:** Rute Ferreira, Cláudia Sousa, Raquel F. S. Gonçalves, Ana Cristina Pinheiro, Mónica Oleastro, Jeroen Wagemans, Rob Lavigne, Ceu Figueiredo, Joana Azeredo, Luís D. R. Melo

**Affiliations:** 1 CEB—Centre of Biological Engineering, University of Minho, 4710-057 Braga, Portugal; rute.ferreira@ceb.uminho.pt (R.F.); claudiasousa@ceb.uminho.pt (C.S.); raquel.goncalves@ceb.uminho.pt (R.F.S.G.); anapinheiro@deb.uminho.pt (A.C.P.); jazeredo@deb.uminho.pt (J.A.); 2 LABBELS—Associate Laboratory, Braga/Guimarães, Portugal; 3 i3S—Instituto de Investigação e Inovação em Saúde, University of Porto, 4200-135 Porto, Portugal; cfigueiredo@ipatimup.pt; 4 Department of Infectious Diseases, National Institute of Health Doctor Ricardo Jorge (INSA), 1649-016 Lisbon, Portugal; Monica.Oleastro@insa.min-saude.pt; 5 Laboratory of Gene Technology, KU Leuven, 3001 Leuven, Belgium; jeroen.wagemans@kuleuven.be (J.W.); rob.lavigne@kuleuven.be (R.L.); 6 Ipatimup—Institute of Molecular Pathology and Immunology of the University of Porto, 4200-135 Porto, Portugal; 7 Department of Pathology, Faculty of Medicine, University of Porto, 4200-319 Porto, Portugal

**Keywords:** *Helicobacter pylori*, prophage, phage therapy, genomic analysis

## Abstract

*Helicobacter pylori*, a significant human gastric pathogen, has been demonstrating increased antibiotic resistance, causing difficulties in infection treatment. It is therefore important to develop alternatives or complementary approaches to antibiotics to tackle *H. pylori* infections, and (bacterio)phages have proven to be effective antibacterial agents. In this work, prophage isolation was attempted using *H. pylori* strains and UV radiation. One phage was isolated and further characterized to assess potential phage-inspired therapeutic alternatives to *H. pylori* infections. HPy1R is a new podovirus prophage with a genome length of 31,162 bp, 37.1% GC, encoding 36 predicted proteins, of which 17 were identified as structural. Phage particles remained stable at 37 °C, from pH 3 to 11, for 24 h in standard assays. Moreover, when submitted to an in vitro gastric digestion model, only a small decrease was observed in the gastric phase, suggesting that it is adapted to the gastric tract environment. Together with its other characteristics, its capability to suppress *H. pylori* population levels for up to 24 h post-infection at multiplicities of infection of 0.01, 0.1, and 1 suggests that this newly isolated phage is a potential candidate for phage therapy in the absence of strictly lytic phages.

## 1. Introduction

*Helicobacter pylori* is a Gram-negative, spiral, and microaerobic bacterium and is considered one of the major human pathogens [1]. It colonizes the human stomach, and in 2017 it was estimated that 4.4 billion individuals worldwide were infected with *H. pylori* [2]. Central Asia and Africa were reported to have the highest prevalence of *H. pylori* infections (>79%), while Oceania had the lowest rate (24.4%) [2]. This bacterium is associated with chronic gastritis, promoting the inflammation and progressive destruction of the architecture and function of the gastric epithelium. More severe infections can progress to peptic ulcers and gastric cancer [3]. The risk factors for *H. pylori* acquisition include low socioeconomic status, lower education levels, unclean drinking water sources, and household crowding [4]. *H. pylori* has the capability of stomach colonization due to the presence of several virulence factors (reviewed in [5]). Among these factors is the urease enzyme, an essential colonization factor [6]. In addition, bacterial shape and flagella are also physical facilitators of bacterial movement within the gastric mucus layer which therefore facilitate the colonization of the gastric mucosa [7,8].

Current treatments for *H. pylori* infection include a set of antibiotics combined with a proton pump inhibitor (PPI), which helps to reduce stomach acidity. The first-line therapeutic strategies recommend the selection of a treatment regimen based on previous antibiotic exposure or on the resistance rate of the region [9]. The decreasing eradication rate (80% to 90% in the 1990s and <70% in recent years), attributable to the increase in the occurrence of antibiotic-resistant strains [9,10], as well as a series of adverse effects promoted by antibiotics [11], have driven the search for alternative therapies or complementary approaches to tackle *H. pylori* infections [12]. In 2017, clarithromycin-resistant *H. pylori* was designated by the World Health Organization (WHO) as a high-priority microorganism for antibiotic research and development [13].

The need for alternative treatments has led researchers to study, among other things, the benefits of using probiotics, natural products, antimicrobial peptides and photodynamic therapy (reviewed in 8). In the last decade, (bacterio)phages have emerged as an effective alternative therapy to fight bacterial infections, mainly those caused by multidrug-resistant (MDR) bacterial strains. Phages are natural predators of bacteria and can be used safely, with minimal disruption to normal flora [14]. Phages usually follow one of two infection cycles: lysogenic (temperate) or strictly lytic (virulent). Temperate phages can integrate into the host genome and reside there dormant as prophages until conditions favour their reactivation [15]. Although strictly lytic phages have generally been preferred for phage therapy purposes, temperate phages have an unexploited potential [16]. They are highly abundant and straightforward to identify and isolate, which is advantageous, especially for fastidious bacteria that require complex growth media. The successful use of temperate phages on fastidious species, such as *Clostridium*, has been reported [17]. In the case of *H. pylori*, the presence of phages in their genomes has previously been shown [18,19,20,21,22,23,24,25,26,27]. While some of these prophages were even induced and studied [19,28,29,30] and virulent *H. pylori* phages have been reported [31,32], the absence of genomic analyses has limited their study. Moreover, the characteristics of *H. pylori* (pro)phages have not yet been well described, which remains the principal hurdle towards the possible application of phage therapy against *H. pylori*.

In this study, we describe a new *H. pylori* phage named HPy1R. HPy1R was isolated after UV radiation treatment from a Portuguese clinical strain and was characterized by its morphology, stability under gastric environmental conditions, efficacy and genomic characteristics.

## 2. Results

Due to the difficulties in isolating *H. pylori* strictly lytic phages and knowing, from previously published studies, that this bacterial species contains prophages within its genome, we attempted to isolate prophages from *H. pylori* strains using UV light. Nineteen strains isolated from gastric biopsies of Portuguese patients were subjected to treatment with UV radiation (Table 1, strains identified with an asterisk (*)). We were able to obtain isolated plaques of one phage from strain 11057A, which was subsequently characterized.

### 2.1. Isolation of a New H. pylori Phage—Morphology and Host Range Analysis

**Figure 1:**
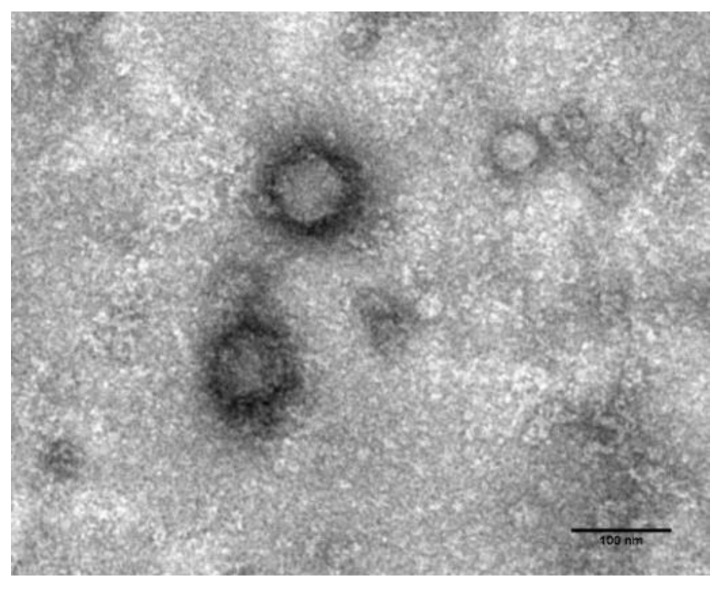
Transmission electron micrographs showing the HPy1R particle morphology, stained with 2% uranyl acetate. Scale bar: 100 nm.

The isolated phage from strain 11057A was named HPy1R. Although it does not form plaques in the host strain, it formed small uniform plaques (1 mm diameter) on 0.4% (*w*/*v*) agar plates of five *H. pylori* strains, one of which (11507) was selected as a propagating strain. Transmission electron microscopy (TEM) images revealed a typical podovirus morphology [33] with a short, non-contractile tail, 17 ± 3 nm (*n* = 9) in length, and with an icosahedral head of 66 ± 6 nm (*n* = 9) (Figure 1).

A panel of 75 random *H. pylori* human clinical isolates from gastric biopsies of Portuguese patients with different gastric diseases and reference strain SS1 were used to determine the phage lytic range and its relative efficiency of plating (EOP, Table 1). Hpy1R displayed a broad spectrum of action, causing visible haloes of inhibition in 78.9% (60/76) of the tested strains. Moreover, HPy1R was able to form plaques in 5 of the 76 strains (6.6%) and the remaining strains were lysed from without (LFW). Fifteen *H. pylori* strains could not be lysed by HPy1R (19.7%) under the growth conditions tested.

### 2.2. Genomic Features of the HPy1R Phage

**Figure 2:**
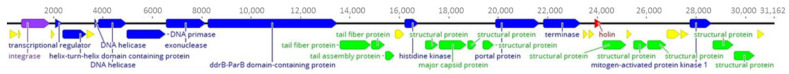
Genome overview of the *Helicobacter* phage HPy1R. Genome map with the predicted 36 CDSs numbered and coloured (blue shows DNA replication and transcription genes, green represents DNA packaging and phage morphogenesis genes, red indicates cells lysis genes, purple indicates integration genes, and yellow shows hypothetical proteins) according to their predicted function. Some important CDSs are highlighted. The nucleotide position (in kb) is indicated above the genome. The figure was generated using Geneious 9.1.4.

Subsequently, the *Helicobacter* phage genome was sequenced using a MiSeq Illumina platform, and after assembly and annotation it was deposited in GenBank with the accession number OM515228. HPy1R has a linear double-stranded DNA (dsDNA) molecule of 31,162 bp with 37.1% GC content. The phage has 287 bp terminal repeat regions at the genome ends. The predicted packaging mechanism was consistent with these direct terminal repeats [34]. The phage genome encodes 36 coding sequences (CDSs), of which 19 could not be functionally assigned, whereas the other 17 were similar to known *H. pylori* phage proteins (Figure 2, Table S1). Interestingly, the majority of the predicted proteins were small in size, with 58% presenting less than 200 residues and only 14% composed of more than 500 amino acids (Table S1). HPy1R genes present a coding density high, with 95.1% of sequences coding and an average of 1.17 genes per 1 Kb. In terms of genome organization, core genes were found to encode proteins related to DNA replication and transcription (e.g., transcriptional regulators, DNA helicase, DNA primase), DNA packaging and morphogenesis (e.g., major capsid proteins, portal protein), cell lysis (e.g., holin), and integration (e.g., integrase).

Across the HPy1R genome, two promoters and one rho-independent terminator were predicted. In addition, no tRNAs were putatively identified.

BLASTn analysis showed that HPy1R is homologous to several *Helicobacter* phages, more specifically *Helicobacter* phage COL 23-PUJ (MW247147; coverage: 80%; identity: 88%). Comparative proteomics showed that HPy1R integrase was very similar to other *H. pylori* prophage integrases (coverage > 90% and identity > 50%, e.g., *Helicobacter* phage Pt22899G, ANT42524.1). In addition, significant sequence similarity with other integrases was detected, including *Nitratiruptor* phage NrS-4 (BCD83183; coverage: 44%; identity: 38%), *Pseudomonas* phage vB_Pae_CF55b (QBI77451; coverage: 61%; identity: 27%) and *Bacillus* phage BtCS33 (YP_006488695; coverage: 71%; identity: 29%). Contrary to observations made for the integrase, the DNA helicase and major capsid protein only showed homology to *Helicobacter* phages.

Based on OrthoVenn2 software analysis, we observed that the COL 23-PUJ, Pt5771G, SwA626G, and pHiHP33 phage genomes share between 66.67% and 97.22% orthologous proteins with HPyR1 (Figure S1). HPy1R therefore belongs to the same phage genus, the *Schmidvirus* in the *Podoviridae* family.

No antibiotic resistance genes or other bacterial-associated virulence determinants were identified in silico with the tools used.

### 2.3. Analysis of Phage Hpy1R Structural Proteins

To confirm the protein composition of the HPy1R phage genome, its structural proteome was analysed. Mass spectrometry (ESI-MS/MS) enabled the identification of 17 proteins, of which 15 had a coverage of over 5% and 16 had more than one unique peptide (Table 2). Among these proteins, eight were structural proteins near the modules of DNA packaging, lysis, and morphogenesis genes, and nine had predicted functions (e.g., tail fibers and major capsid proteins), putatively involved in DNA packaging, morphogenesis functions, and integration.

### 2.4. HPy1R Phage Shows Stability at Gastric Environmental Conditions

**Figure 3:**
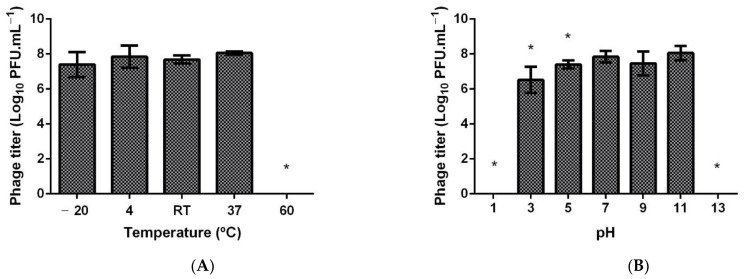
Thermal and pH stability of the HPy1R phage. (**A**) Thermal assays were performed under different temperatures (−20 °C, 4 °C (control), room temperature (25 °C), 37 °C, and 60 °C) for 24 h. (**B**) pH assays were performed under different pH levels (1, 2, 3, 5, 7 (control), 9, 11, 13), for 24 h at room temperature. Error bars represent standard deviations for three independent assays (*n* = 3) performed in duplicate. * Statistical differences compared with the control (*p* < 0.05).

The HPy1R (10^8^ PFU mL^−^^1^) was assessed in vitro for 24 h phage stability under different temperatures and pH conditions. Notably, the results showed that in a temperature range of −20 °C to 37 °C and at pH levels of 7, 9, and 11 (Figure 3) phage viability was not impacted. At pH 3.0 and 5.0, the PFU counts decreased by 1.34 and 0.45 orders of magnitude, respectively (*p* < 0.05) (Figure 3B). The evaluation of phage viability at 60 °C, pH 1 and 13 revealed a total loss of phage titer.

**Figure 4:**
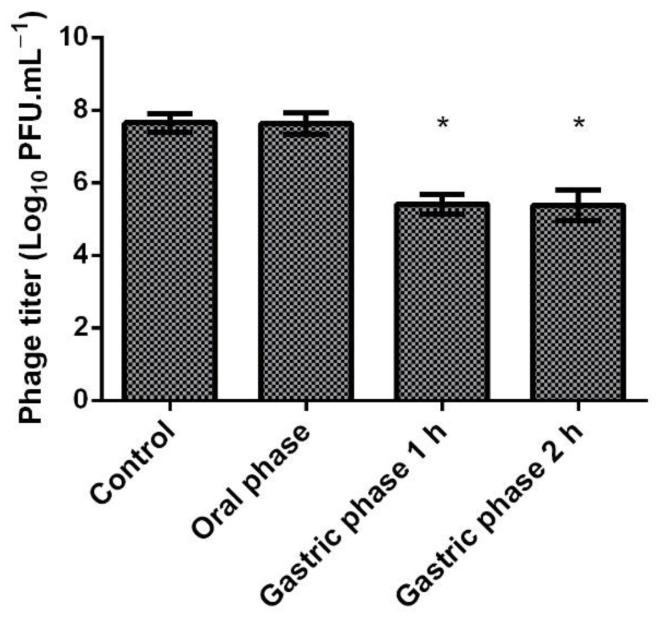
Stability of the HPy1R after the in vitro digestion. Error bars represent standard deviations for three independent assays (*n* = 3) performed in duplicate. * Statistical differences compared with the control (*p* < 0.05).

In addition, to study the gastric behaviour of HPy1R, a harmonized static in vitro digestion model comprising oral and gastric phases was set up [35]. After a meal, the acidity of the gastric contents is buffered by proteins and remains around pH 3 for approximately 90 min [36]. In vitro methods aim to mimic physiological conditions in vivo, taking into account the presence of digestive enzymes and their concentrations, pH, digestion time, and salt concentrations, among other factors. Moreover, they have the advantage of being more rapid, less expensive, less labour-intensive, and not being subject to ethical restrictions [35]. Under this model, HPy1R proved to be stable in the 2 min of oral phase, where no loss of titer was observed compared to the control (Figure 4). Moreover, in the gastric phase, the phage concentration decreased by 2.24 and 2.27 orders of magnitude after 1 and 2 h, respectively, relative to the control, without simulated salivary and gastric fluids (*p* < 0.05) (Figure 4).

### 2.5. Assessing the Phage HPy1R Potential for H. pylori Control

**Figure 5:**
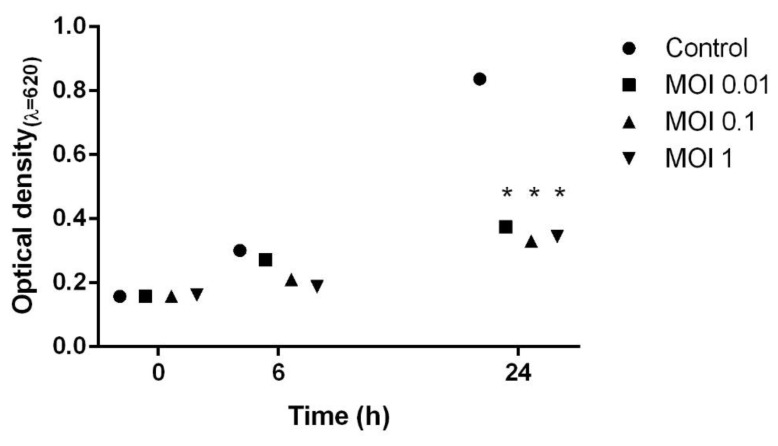
*H. pylori* infected with HPy1R phages at different MOIs. Infection assays were performed for 24 h at 37 °C, 120 rpm under microaerophilic conditions at the indicated MOIs. * Statistical differences compared with the control (*p* < 0.05).

The efficacy of HPy1R against a culture of *H. pylori* was tested. In vitro tests demonstrated that the phage proved to be less effective 6 h after infection, as no statistical differences in the number of bacterial cells between control and phage-treated cells could be observed. HPy1R could maintain the *H. pylori* population at low levels for up to 24 h post-infection, with multiplicities of infection (MOIs) of 0.01, 0.1, and 1 (Figure 5). Interestingly, similar growth inhibition in the bacterial cell population was observed using all tested MOIs.

## 3. Discussion

Driven by the advantages and potential of phage therapy, associated with the lack of exploration of the phage field with respect to *H. pylori*, we present in this work the isolation of HPy1R and the first complete characterization of an *H. pylori* phage for a future therapeutic approach. HPy1R was isolated by exposing the clinical *H. pylori* strain 11057A to UV radiation for 60 s. With a similar protocol, Lehours et al. [19] also reported the use of UV light as an agent to isolate phages. Nevertheless, spontaneous induction of phages was also observed in other *H. pylori* strains [29,30,32,37], as well as induction of prophages after subsequent exposure to citrate–phosphate buffer at pH 6 and 3 [38]. To date, there have been no published reports on *H. pylori* prophages induced using Mitomycin C (reviewed in [12]).

Of the 75 *H. pylori* clinical-strains used in this work, the HPy1R phage was only able to form plaques in five of them, with strain 11507 being selected as the propagation strain. The other strains were either immune or resistant to the phage. Superinfection immunity is typically associated with lysogeny. With immunity, bacteria prevent them from being infected by two or more related prophages [39]. Prophages are quite common in *H. pylori* genomes; however, as there are no genomic data on the clinical strains used in this work, we cannot say with certainty that this is a case of superinfection immunity.

Regarding morphology, the phage has the typical characteristics of the *Podoviridae* family, similar to *H. pylori* 1961P and ΦHPE1 phages [29,31]. The phages KHP30 and KHP40 reported by Uchiyama et al. [30] belong to the *Corticoviridae* family, and phages HP1, PhiHp33, and ΦHPE2 appeared to be morphology compatible with siphophages [19,28,31].

HPy1R showed a broad spectrum of activity (60 out of 76 strains) and was able to replicate in five strains out of 76 tested (6.6%). A limited capacity to replicate in different strains was previously observed for other reported *H. pylori* phages. Siphovirus HP1 only showed a reduction of opacity in two out of 10 strains tested (20%), and podovirus 1961P was only able to form single plaques in two out of 48 *H. pylori* tested strains tested (4%), despite being able to form clear zones in all 44 strains of *H. pylori* [28,29]. In contrast, the *H. pylori* phage KHP30, belonging to the *Tectiviridae* family, showed the ability to form plaques in 28 of the 44 strains tested (63.6%) [30]. Although not proven, the hypothesis that phage morphology may be related to a phage’s ability to infect strains could be an explanation for the differences in the lytic spectra of *H. pylori* phages. In *Klebsiella pneumoniae*, *Podoviridae,* and *Siphoviridae,* viruses exhibited a narrower lytic spectrum of activity when 32 phages belonging to the *Caudovirales* were evaluated for lytic activity on 254 bacterial strains [40]. The ability of each phage to infect a specific strain is due to a combination of factors, including host-binding protein specificity, biochemical interactions during infection, mechanisms of resistance to bacterial phages, and the presence of plasmids and prophages [41,42]. A review of bacteriophage resistance mechanisms identified superinfection immunity, superinfection exclusion (Sie) systems, restriction–modification systems, and CRISPR-associated (*cas*) genes as bacterial strategies to combat these viruses [43]. The presence of prophages in the genome of strains chosen for the lytic spectrum may also be the reason for the limited HPy1R host range. However, to prove this, a complementary analysis of all the genomes of clinical isolates would be necessary. In the lytic spectrum, the use of the *H. pylori* SS1 strain should also be highlighted. This strain has become a field standard in *H. pylori* mouse infections [44]. Lamentably, phage HPy1R was unable to infect this strain. This could be a consequence of the adaptation of the bacteria to mice and the fact that the phage was isolated from a human clinical strain. On the other hand, this result may increase future problems in more specific studies involving mouse models and phages (e.g., infection and immunity studies), since there is a difficulty in finding *H. pylori* strains that colonize mice [45].

To better characterize the isolated phage, we sequenced its genome. The HPy1R genome size has a slightly larger genomic size compared to previously sequenced *H. pylori* phages [19,29]. In addition to being an induced phage, the temperate nature of the phage was also confirmed by the detection of integrase in the host genome (gp 3, Table S1). Furthermore, as the HPy1R is terminus type short exact direct repeat end, generalized transduction was not identified, unlike phages characterized as headful packaging [34]. In this last terminus type, the lack of terminase sequence specificity allows host DNA near the site of phage DNA integration to be transduced, and therefore, therapeutically, this phage-type should be avoided, due to the facility of transducing resistance genes [34].

We show that HPy1R has a significant genomic similarity (80%) to phage COL 23-PUJ (Figure S1). However, it should be noted that this phage has been identified in an *H. pylori* strain as a prophage and was never induced [18]. The similarity to previously sequenced phages shows that HPy1R has all the features necessary for inclusion in the *Schmidvirus* genus of the *Podoviridae* family. Proteins derived from the phage particles were analysed by mass spectrometry, and 17 structural proteins were confirmed (Table 2). No tRNAs or antibiotic resistance genes were identified with the used tools. However, gp 7, classified as a hypothetical protein, showed greater similarity to a previously identified putative ATP-binding-cassette (ABC), a gene associated with virulence and resistance to antibiotics. ABC transporters are widespread among living organisms. In bacteria, they predominantly act in the uptake of molecules as opposed to efflux [46]. The relationship between ABC transporters and the virulence of pathogenic bacteria is associated with the need to capture nutrients in order to adapt to environmental conditions. ABC importers have previously been identified in pathogenic species, such as *Staphylococcus aureus*, *Campylobacter jejuni,* and *Acinetobacter baumannii*, and have been shown to be critical to the virulence of these bacteria (reviewed in [47]). Knowledge of the role of ABC transporters in bacterial virulence has enabled the study of antibacterial therapies that inhibit these transporters [48,49].

Considering the lytic cassette when analysing the genome of HPy1R, only holin was identified (gp 26, Table S1) Therefore, further studies will be necessary to identify endolysins and explore their antibacterial potential.

Despite the numerous advantages of phages, temperature and pH are recognized as external physical factors that influence phage adsorption and the ejection of genetic material, along with multiplication, stability, and viability [50]. In this work, we explored the stability of *H. pylori* phages at different temperatures and pH values for the first time and then applied them in a static in vitro digestion model comprising oral and gastric phases [35]. Phage HPy1R was demonstrated to be stable at 37 °C and pH 3, which are typical conditions found in the gastric environment. Similarly, the KHP30 phage also showed stability over a pH range between 2.5 and 10 [30]. In addition, in the oral phase, no loss of titer in the HPy1R phage was observed when compared to the control (Figure 4). On the other hand, in the gastric phase, the phage concentration decreased compared to the oral phase. A small loss in phage titer had already been observed in the pH stability tests, where at pH 3 the titer decreased by 1.34 orders of magnitude (Figure 3). The presence of salts and enzymes in gastric juices can reduce the proliferation and concentration of phages, altering their biological and structural components [51]. The loss of phage titer in the gastric phase in the in vitro assay may probably be related to this fact. However, phage encapsulation could be a viable solution to overcome the adverse conditions of the gastric environment [32,52]. Another possibility is the natural coating of the phages through genetic manipulation. Using Bacteriophage Recombineering of Electroporated DNA (BRED), it was possible to display phospholipids on the surface of T7 phages, improving their stability without affecting their ability to infect [51].

In the present study, the efficacy of phage HPy1R in combating *H. pylori* infection was also explored. We observed that the presence of HPy1R phages was able to keep *H. pylori* populations at low levels for up to 24 h after infection with MOIs of 0.1 and 1 (Figure 5). However, there were not enough data to determine whether the results for the different MOIs were associated with increased rates of lysogeny. More studies should be undertaken in the future to see whether HPy1R phage lysogeny can occur in the propagating strain (11507). Due to its being a fastidious bacterium, *H. pylori* has a slow growth rate. We can hypothesize that phage replication is quicker than the bacterial doubling time such that even low concentrations of phage can control bacterial growth. With *H. pylori* phage Hp ϕ, the phage efficacy was also independent of *H. pylori* MOI [32]. An efficacy assay with AGS human gastric cells infected with a clinical *H. pylori* strain and treated with phage Hp ϕ immediately and after 24 h post-infection showed a decrease in bacterial cell count of about 3 and 1.5 orders of magnitude, respectively, at 3 h of treatment [32].

These results suggest that phages may be a realistic alternative to combat *H. pylori* infections. However, additional studies are needed, including regarding HPy1R safety, toxicity, and the (absence of) transmission of virulence genes. With the currently available genetic engineering tools and resources, the efficacy and safety of phages can be improved, for example, via the removal of virulence genes. Furthermore, advances in synthetic biology offer opportunities for creating lytic and customized variants of temperate phages [16]. Another option for broadening the spectrum of action of phages is the use of phage cocktails in the treatment of *H. pylori* infections [53].

## 4. Materials and Methods

### 4.1. Bacterial Strains and Growth Conditions

The *H. pylori* strains used in this study were isolated from human gastric biopsies and belong to the collection of bacterial strains from INSA—National Institute of Health Doctor Ricardo Jorge, Lisbon, Portugal. Strain SS1 was a kind gift from Professor James Fox, Massachusetts Institute of Technology, Cambridge, MA, USA.

Bacteria were cultured on liquid TSB (VWR, Radnor, PA, USA) medium supplemented with 10% FBS (Biochrom, Cambridge, UK), pH 7.0 ± 0.2, or in solid *H. pylori* selective medium (Columbia Blood Agar (Thermo Scientific Oxoid, Basingstoke, UK), Horse Blood Defibrinated (Thermo Scientific Oxoid, Basingstoke, UK), and *Helicobacter pylori* selective supplement (Dent, Thermo Scientific Oxoid, Basingstoke, UK), pH 7.0 ± 0.2 at 37 °C, under microaerophilic conditions (10% CO_2_, 5% O_2_). For phage propagation, solid plates (1.2% agar) and top agar (0.4% agar) of NZCYM broth (Sigma-Aldrich, St. Louis, MO, USA), pH 7.0 ± 0.2, were used.

### 4.2. Prophage Isolation and Production

For prophage isolation, 3 mL of *H. pylori* cells (Table 1) (OD_620nm_ ≈ 0.2) were centrifuged at 6000× *g* for 10 min and the pellet was resuspended in 3 mL of sterile 0.1 M MgSO_4_ (Sigma-Aldrich, St. Louis, MO, USA). The suspensions were then transferred to a sterile 12-well plate and irradiated with a UV 254 nm lamp for 30, 60, 90, and 120 s, at a distance of 12 cm. After UV treatment, 500 µL of the culture was transferred to 4.5 mL of TSB + 10% FBS followed by an additional 24 h of incubation in microaerophilic conditions. Afterwards, the culture was centrifuged (3000× *g*, 12 min, 4 °C) and the supernatant was filtered using a 0.22 µm polyethersulfone (PES) filter. Spot assays against host–bacterial lawns and *H. pylori* strain 11507 were performed to check for the presence of phages. Inhibition haloes picking was performed until plaque morphology was observed. The diameters of six phage plaques were measured and characterized.

Phage production was carried out using the double-layer agar method previously described, with some modifications [54]. A volume of 100 µL of phage solution was spread in a *H. pylori* strain 11507 lawn using a strip of paper. Petri dishes were incubated for 2 to 3 days at 37 °C and under microaerophilic conditions. After complete lysis, 2 mL of SM buffer (5.8 g L^−1^ NaCl (Thermo Fisher Scientific, Waltham, MA, USA), 2 g L^−1^ MgSO_4_.7H_2_O (PanReac AppliChem, Darmstadt, Germany), 50 mL L^−1^ 1 M Tris-base (Thermo Fisher Scientific, Waltham, MA, USA), pH 7.5, 0.002% (*w*/*v*) gelatin (Sigma-Aldrich, St. Louis, MO, USA)) were added to each Petri dish. Plates were further incubated at 4 °C, 50–90 rpm, for 7 h. Subsequently, the liquid and top agar were collected and centrifuged (10 min, 10,000× *g*, 4 °C), and the supernatant was filtered as described above. The phage was stored at 4 °C until use.

### 4.3. Transmission Electron Microscopy

Phage morphology was observed by TEM as previously described [55]. Briefly, phage particles were collected after centrifugation (25,000× *g*, 4 °C, 1 h). The pellet was washed twice with tap water before centrifugation. Furthermore, phage was deposited on copper grids with carbon-coated Formvar films, stained with 2% uranyl acetate (pH 4), and analysed using a Jeol JEM 1400 transmission electron microscope.

### 4.4. DNA Isolation and Genome Sequencing and Annotation

Phage total DNA was extracted using the phenol–chloroform protocol described by [55], with some modifications. Phage lysate was treated with 12.5 μL mL^−^^1^ of MgCl_2_ (1 M, Sigma-Aldrich, St. Louis, MO, USA), 1 μL mL^−^^1^ of DNAse I (10 mg mL^−^^1^, Thermo Fisher Scientific, Waltham, MA, USA), and 1 μL mL^−^^1^ of RNAse A (100 mg mL^−^^1^, VWR, Radnor, PA, USA) for 1 h at room temperature. Phage structural proteins were digested overnight at 56 °C in the presence of 50 µg proteinase K ml^−^^1^, 20 mM EDTA (Thermo Fisher Scientific, Waltham, MA, USA) and 1% SDS (Thermo Fisher Scientific, Waltham, MA, USA). Phage DNA was extracted in phenol (PanReac AppliChem, Darmstadt, Germany) – chloroform (Merck, Darmstadt, Germany) (1:1) and, after centrifugation (13,000× *g*, 10 min, 4 °C), the supernatant was purified with one volume of chloroform. DNA was then precipitated with 0.8 volumes of ethanol (Thermo Fisher Scientific, Waltham, MA, USA) and 0.2 volumes of 3 M sodium acetate (pH 4.6) and centrifuged (10 min, 7600× *g*, 4 °C). The resulting pellets were air-dried and resuspended in deionized distilled water.

For sequencing analysis, a DNA library was constructed using the Illumina Nextera XT library preparation kit. Reads were demultiplexed and de novo assembled into a single contig with an average coverage above 100× using Geneious R9 and manually inspected. MyRAST [56] and tRNAscan-SE [57] were used to determine the ORFs and tRNAs, respectively.

For similarity search and structured prediction, proteins were analysed using BLASTp [58] and HHpred [59]. TMHMM [60], Phobius [61], HMMTOP [62], and SignalP [63] servers were used to predict transmembrane domains and to identify possible signal peptide cleavage sites. Putative promoter regions were analysed using PromoterHunter from phiSITE [64] and Promoter 2.0 [65]. The energy was calculated using Mfold [66], and ARNold [67] was used to predict factor-independent terminators. Comparative genomic and proteomic analyses were performed with BLASTn or OrthoVenn [68]. The Resistance Gene Identifier (RGI) of CARD (the Comprehensive Antibiotic Resistance Database) [69], with a display of results with perfect, strict, and loose hits, was used to check the presence of antibiotic resistance in the phage genome. Further, the TAfinder [70] tool was used for the prediction of toxins. The packaging mechanisms and genome termini were determined using PhageTerm [71].

### 4.5. Liquid Chromatography–Tandem Mass Spectrometry (LC–MS/MS) Analysis

Phage structural proteins were extracted by methanol–chloroform extraction, as described previously [72], followed by in-gel trypsinization [73]. Eluted peptide mixtures were then dried and re-dissolved in 20 µL loading solvent (0.1% trifluoroacetic acid in water–acetonitrile (98:2, *v*/*v*)), of which 10 µL was injected for LC–MS/MS analysis on an Ultimate 3000 RSLC nano-LC (Thermo Fisher Scientific, Waltham, MA, USA) in-line coupled to a Q Exactive mass spectrometer (Thermo Fisher Scientific, Waltham, MA, USA).

Peptides were first loaded on a trapping column made in-house (100 μm internal diameter (I.D.) × 20 mm, 5 μm beads C18 Reprosil-HD, Dr. Maisch, Ammerbuch-Entringen, Ammerbuch, Germany) and, after flushing from the trapping column, peptides were separated on a 50 cm µPAC™ column with C18-endcapped functionality (Pharmafluidics, Ghent, Belgium) and kept at a constant temperature of 50 °C. Peptides were eluted by a stepped gradient from 98% solvent A (0.1% formic acid in water) to 30% solvent B (0.1% formic acid in water–acetonitrile, 20/80 (*v*/*v*)) in 135 min up to 50% solvent B in 26 min, followed by a 6 min wash reaching 95% solvent B, all at a stepped flow rate starting from 750 nL/min for 9 min to 300 nL/min, until the end of the run.

The mass spectrometer was operated in data-dependent, positive ionization mode, automatically switching between MS and MS/MS acquisition for the five most abundant peaks in a given MS spectrum. The source voltage was 4.2 kV, and the capillary temperature was 275 °C. One MS1 scan (*m*/*z* 400–2000, AGC target 3 × 10^6^ ions, maximum ion injection time 80 ms), acquired at a resolution of 70,000 (at 200 *m*/*z*), was followed by up to five tandem MS scans (resolution 17,500 at 200 *m*/*z*) of the most intense ions fulfilling predefined selection criteria (AGC target 50.000 ions, maximum ion injection time 80 ms, isolation window 2 Da, fixed first mass 140 *m*/*z*, spectrum data type: centroid, intensity threshold 1.3 × 10^4^, exclusion of unassigned, 1, 5–8, >8 positively charged precursors, peptide match preferred, exclude isotopes on, dynamic exclusion time 15 s). The HCD collision energy was set to 25% Normalized Collision Energy and the polydimethylcyclosiloxane background ion at 445.120025 Da was used for internal calibration (lock mass). QCloud was used to control instrument longitudinal performance during the run [74].

LC–MS/MS runs of both samples were searched separately using the MaxQuant algorithm (version 2.0.2.0) using default search settings, including a false discovery rate set at 1% at peptide and protein levels, trypsin-specific cleavage with a maximum of two missed cleavages, carbamidomethylation on cysteine residues as a fixed modification, and oxidation on methionines as variable modification. Spectra were searched against the annotated phage proteomes.

### 4.6. Host Range Analysis and Efficiency of Plating Determination

The host range of phage HPy1R was determined as previously described, with some modifications [17]. A total of 76 strains (clinical isolates from patients with different gastric diseases and the SS1 mouse reference strain) were selected to determine host range (Table 1). Bacterial lawns were formed on NZCYM broth plates by adding 500 μL of bacterial culture (OD_620nm_ > 0.7) of each strain to be tested. A 10 µL drop of serial tenfold dilutions of *H. pylori* phage was then spotted onto each bacterial lawn and the Petri dishes were incubated for 2 to 3 days at 37 °C under microaerophilic conditions. EOP is presented in PFU mL^−^^1^. EOP negative was scored as 0. LFW represents lysis from without.

### 4.7. Stability in the Gastric Environment

Thermal and pH stability tests were performed as previously described [75]. Thermal stability was determined by incubating 10^8^ PFU mL^−^^1^ of *H. pylori* phage at different temperatures: −20 °C, 4 °C (control), room temperature (25 °C), 37 °C, and 60 °C, for 24 h. Similarly, the effect of pH was evaluated using a universal pH buffer (150 mM KCl (Sigma-Aldrich, St. Louis, MO, USA), 10 mM KH_2_PO_4_ (Panreac AppliChem, Darmstadt, Germany), 10 mM Na_3_C_6_H_5_O_7_, (Thermo Fisher Scientific, Waltham, MA, USA), and 10 mM H_3_BO_3_ (Thermo Fisher Scientific, Waltham, MA, USA), adjusted to different pH values, i.e., 1, 2, 3, 5, 7 (as control), 9, 11, and 13, at room temperature for 24 h. In both experiments, phage was diluted and plated on *H. pylori* propagation host lawns for enumeration. Averages and standard deviations for all experiments are given for *n* = 3 repeats.

To simulate the oral and gastric phases, a stability test of the isolated phage was also performed using the harmonized INFOGEST in vitro digestion model according to the procedure reported by Minekus et al. [35]. A phage suspension of 10^8^ PFU mL^−^^1^ was subjected to oral digestion with an equal volume of simulated salivary fluid (SSF, composed of KCl 15.1 mmol L^−^^1^, KH_2_PO_4_ 3.7 mmol L^−^^1^, NaHCO_3_ 13.6 mmol L^−^^1^, MgCl_2_(H_2_O)_6_ 0.15 mmol L^−^^1^, (NH_4_)_2_CO_3_ 0.06 mmol L^−^^1^, and HCl 1.1 mmol L^−^^1^), CaCl_2_(H_2_O)_2_ 0.3 mol L^−^^1^ (to achieve 0.75 mmol L^−^^1^ at the final mixture), and Milli-Q water (to the final volume). The pH of the resulting mixture was set to 7.0 and the oral phase simulation was carried out at 37 °C, while shaking at 120 rpm for 2 min. Note that α-amylase was not added in case the samples did not contain starch. The gastric phase was simulated by adding to the previous mix simulated gastric fluid (SGF, composed of KCl 6.9 mmol L^−^^1^, KH_2_PO_4_ 0.9 mmol L^−^^1^, NaHCO_3_ 25 mmol L^−^^1^, NaCl 47.2 mmol L^−^^1^, MgCl_2_(H_2_O)_6_ 0.12 mmol L^−^^1^, (NH_4_)_2_CO_3_ 0.5 mmol L^−^^1^, and HCl 15.6 mmol L^−^^1^) at a ratio of 1:1 (*v*/*v*), porcine pepsin (at a concentration of 2000 U/mL in the final mixture), and CaCl_2_(H_2_O)_2_ 0.3 mol L^−^^1^ (at a concentration of 0.075 mmol L^−^^1^ in the final mixture). The pH was adjusted to 3.0 with HCl (1 mol L^−^^1^) and Milli-Q water was added to make up the final volume. The incubation was carried out under the same conditions used previously for 120 min. After each digestion phase and 1 h after the gastric digestion phase, a sample was taken for phage enumeration. Averages and standard deviations for all experiments are given for *n* = 3 repeats.

### 4.8. Phage Infection Assay

*H. pylori* (OD_620nm_ ≈ 0.200) was incubated at 37 °C, 120 rpm under microaerophilic conditions, with or without the addition of phage at different MOIs (0.01, 0.1, and 1). Samples were collected at 6 and 24 h and the OD_620nm_ was measured. Averages and standard deviations for all experiments are given for *n* = 3 repeats.

### 4.9. Statistical Analysis

In all the assays, averages and standard deviations were determined based on 3 independent experiments (*n* = 3) performed in duplicate. Statistical analysis was carried out using two-way repeated-measures analysis of variance (ANOVA) with Bonferroni post hoc tests using GraphPad Prism 6. Differences between samples were considered significant at a *p*-value of 0.05 or less.

## 5. Conclusions

In summary, a new phage against *H. pylori*, named HPy1R, was identified. In the absence of strictly lytic *H. pylori* phages, the characteristics and properties of HPy1R indicate that we are at the beginning of the study of the use of phage therapy in the control of infections by *H. pylori*. Furthermore, HPy1R remained stable over a wide range of pHs and temperatures, and, despite the small loss observed in the in vitro gastric phase, the results seem to be encouraging regarding the prediction of the stability of this phage in the stomach.

With increasing resistance to antibiotics, phages and their lysis proteins have shown good indicators in the search for alternative therapies. Here, we have provided further evidence supporting the therapeutic potential of phages against infections caused by *H. pylori*, one of the major human gastric pathogens. The discovery of new and well-characterized phages, as well as the identification and characterization of endolysins, against *H. pylori* will pave the way for the use of phage therapy as an alternative strategy to control *H. pylori* infections.

## Figures and Tables

**Table 1:** HPy1R lytic spectra and efficiency of plating (EOP) against different *H. pylori* strains. Strains subjected to UV radiation treatment are identified with an asterisk (*). EOP is presented in PFU mL^−^^1^. EOP negative was scored as 0. LFW represents lysis from without.

Strains	Origin	Infectivity	EOP (PFU mL^−^^1^)
11061 *	Human gastric biopsy	−	0
11538AIC *	Human gastric biopsy	+	LFW
11417 *	Human gastric biopsy	−	0
11470 *	Human gastric biopsy	+	LFW
11444 *	Human gastric biopsy	−	0
11507 *	Human gastric biopsy	+	4.50 × 10^8^
11471 *	Human gastric biopsy	+	1.10 × 10^5^
11508 *	Human gastric biopsy	+	6.50 × 10^6^
11509 *	Human gastric biopsy	+	LFW
11512 *	Human gastric biopsy	+	LFW
11524 *	Human gastric biopsy	+	2.60 × 10^6^
11414 *	Human gastric biopsy	+	LFW
11515 *	Human gastric biopsy	+	LFW
11438A *	Human gastric biopsy	+	LFW
11057A *	Human gastric biopsy	−	0
11423 *	Human gastric biopsy	−	0
11537AIC *	Human gastric biopsy	+	LFW
11525 *	Human gastric biopsy	+	LFW
11532 *	Human gastric biopsy	+	LFW
11468	Human gastric biopsy	+	LFW
11046	Human gastric biopsy	+	LFW
11058	Human gastric biopsy	+	4.20 × 10^6^
11421	Human gastric biopsy	+	LFW
11402	Human gastric biopsy	+	LFW
11406	Human gastric biopsy	+	LFW
11068	Human gastric biopsy	+	LFW
11053	Human gastric biopsy	−	LFW
11426	Human gastric biopsy	−	0
11521AIC	Human gastric biopsy	+	LFW
11411	Human gastric biopsy	−	0
11410	Human gastric biopsy	+	LFW
11413	Human gastric biopsy	+	LFW
11422	Human gastric biopsy	+	LFW
11062A	Human gastric biopsy	+	LFW
11400	Human gastric biopsy	+	LFW
11446	Human gastric biopsy	+	LFW
11054A	Human gastric biopsy	−	0
11402	Human gastric biopsy	+	LFW
11401	Human gastric biopsy	+	LFW
11415	Human gastric biopsy	+	LFW
11425	Human gastric biopsy	+	LFW
11405	Human gastric biopsy	−	0
11063A	Human gastric biopsy	+	LFW
11416A	Human gastric biopsy	−	0
11403A	Human gastric biopsy	+	LFW
11069	Human gastric biopsy	+	LFW
11419	Human gastric biopsy	+	LFW
11404A	Human gastric biopsy	+	LFW
H6	Human gastric biopsy	+	LFW
H7	Human gastric biopsy	+	LFW
H11	Human gastric biopsy	+	LFW
11004	Human gastric biopsy	+	LFW
11029	Human gastric biopsy	+	LFW
11016A	Human gastric biopsy	−	0
11025	Human gastric biopsy	+	LFW
11523AIC	Human gastric biopsy	+	LFW
11469A	Human gastric biopsy	+	LFW
11514AIC	Human gastric biopsy	+	LFW
11427	Human gastric biopsy	−	0
11437	Human gastric biopsy	+	LFW
11466A	Human gastric biopsy	+	LFW
11530	Human gastric biopsy	+	LFW
11440	Human gastric biopsy	−	0
11467A	Human gastric biopsy	+	LFW
11458	Human gastric biopsy	+	LFW
11474	Human gastric biopsy	+	LFW
11418A	Human gastric biopsy	+	LFW
11436	Human gastric biopsy	+	LFW
11137A/C	Human gastric biopsy	+	LFW
11513	Human gastric biopsy	+	LFW
11441	Human gastric biopsy	+	LFW
11517	Human gastric biopsy	+	LFW
11519	Human gastric biopsy	+	LFW
11439	Human gastric biopsy	−	0
11511	Human gastric biopsy	+	LFW
ATCC SS1	Mouse (B cell); mouse (myeloma)	−	0

**Table 2:** Bacteriophage HPy1R proteins identified by ESI-MS/MS. The SDS–PAGE gel bands in which the proteins were identified are indicated as well as protein mass, the number of identified unique peptides, and the protein sequence that is covered by the peptide (in %).

Protein	Putative Function	Number of Unique Peptides	Sequence Coverage (%)	Protein MW (kDa)
gp 3	Integrase	1	2.8	46.032
gp 12	Ddrb-parb domain-containing protein	64	43.8	199.56
gp 13	Tail fiber protein	21	63.3	48.128
gp 14	Tail fiber protein	5	19.6	20.823
gp 17	Histidine kinase	3	27.2	22.384
gp 18	Structural protein	3	22.8	21.589
gp 19	Major capsid protein	32	87.5	41.52
gp 20	Structural protein	8	77.4	13.421
gp 21	Structural protein	6	44.2	16.42
gp 22	Portal protein	17	37.8	69.502
gp 23	Terminase	2	3.3	60.627
gp 27	Structural protein	15	47.1	37.867
gp 29	Structural protein	8	42.5	20.899
gp 30	Structural protein	5	37.7	29.573
gp 33	Mitogen-activated protein kinase 1	10	30.2	35.25
gp 34	Structural protein	13	48.6	32.682
gp 35	Structural protein	2	7.3	32.537

## Data Availability

The data presented in this study are available on request from the corresponding author.

## Supplementary Materials

The following supporting information can be downloaded at: https://www.mdpi.com/article/10.3390/ijms23147885/s1.

## References

[1] Camilo V., Sugiyama T., Touati E. (2017). Pathogenesis of *Helicobacter pylori* infection. Helicobacter.

[2] Hooi J.K.Y., Lai W.Y., Ng W.K., Suen M.M.Y., Underwood F.E., Tanyingoh D., Malfertheiner P., Graham D.Y., Wong V.W.S., Wu J.C.Y. (2017). Global Prevalence of *Helicobacter pylori* Infection: Systematic Review and Meta-Analysis. Gastroenterology.

[3] Miftahussurur M., Yamaoka Y., Graham D.Y. (2017). *Helicobacter pylori* as an oncogenic pathogen, revisited. Expert Rev. Mol. Med..

[4] Mezmale L., Coelho L.G., Bordin D., Leja M. (2020). Review: Epidemiology of *Helicobacter pylori*. Helicobacter.

[5] Denic M., Touati E., De Reuse H. (2020). Review: Pathogenesis of *Helicobacter pylori* infection. Helicobacter.

[6] Ansari S., Yamaoka Y. (2017). Survival of *Helicobacter pylori* in gastric acidic territory. Helicobacter.

[7] Martínez L.E., Hardcastle J.M., Wang J., Pincus Z., Tsang J., Hoover T.R., Bansil R., Salama N.R. (2016). *Helicobacter pylori* strains vary cell shape and flagellum number to maintain robust motility in viscous environments. Mol. Microbiol..

[8] Sycuro L.K., Pincus Z., Gutierrez K.D., Biboy J., Stern C.A., Vollmer W., Salama N.R. (2010). Peptidoglycan crosslinking relaxation promotes *Helicobacter pylori*’s helical shape and stomach colonization. Cell.

[9] Malfertheiner P., Megraud F., O’Morain C.A., Gisbert J.P., Kuipers E.J., Axon A.T., Bazzoli F., Gasbarrini A., Atherton J., Graham D.Y. (2017). Management of *Helicobacter pylori* infection-the Maastricht V/Florence Consensus Report. Gut.

[10] Savoldi A., Carrara E., Graham D.Y., Conti M., Tacconelli E. (2018). Prevalence of Antibiotic Resistance in *Helicobacter pylori*: A Systematic Review and Meta-analysis in World Health Organization Regions Materials and Methods Search Strategy and Selection Criteria Data Extraction. Gastroenterology.

[11] Lopes D., Nunes C., Martins M.C.L., Sarmento B., Reis S. (2014). Eradication of *Helicobacter pylori*: Past, present and future. J. Control. Release.

[12] Sousa C., Ferreira R., Azevedo N.F., Oleastro M., Azeredo J., Figueiredo C., Melo L.D.R. (2021). *Helicobacter pylori* infection: From standard to alternative treatment strategies. Crit. Rev. Microbiol..

[13] Tacconelli E., Carrara E., Savoldi A., Harbarth S., Mendelson M., Monnet D.L., Pulcini C., Kahlmeter G., Kluytmans J., Carmeli Y. (2018). Discovery, research, and development of new antibiotics: The WHO priority list of antibiotic-resistant bacteria and tuberculosis. Lancet Infect. Dis..

[14] Loc-Carrillo C., Abedon S.T. (2011). Pros and cons of phage therapy. Bacteriophage.

[15] Salmond G.P.C., Fineran P.C. (2015). A century of the phage: Past, present and future. Nat. Rev. Microbiol..

[16] Monteiro R., Pires D.P., Costa A.R., Azeredo J. (2019). Phage Therapy: Going Temperate?. Trends Microbiol..

[17] Meader E., Mayer M.J., Steverding D., Carding S.R., Narbad A. (2013). Evaluation of bacteriophage therapy to control Clostridium difficile and toxin production in an in vitro human colon model system. Anaerobe.

[18] Muñoz A.B., Trespalacios-Rangel A.A., Vale F.F. (2021). An American lineage of *Helicobacter pylori* prophages found in Colombia. Helicobacter.

[19] Lehours P., Vale F.F., Bjursell M.K., Melefors O., Advani R., Glavas S., Guegueniat J., Gontier E., Lacomme S., Alves Matos A. (2011). Genome sequencing reveals a phage in *Helicobacter pylori*. MBio.

[20] Vale F.F., Vadivelu J., Oleastro M., Breurec S., Engstrand L., Perets T.T., Mégraud F., Lehours P. (2015). Dormant phages of *Helicobacter pylori* reveal distinct populations in Europe. Sci. Rep..

[21] Secka O., Vale F.F., Buissonnière A., Thomas J.E., Mégraud F., Lehours P. (2017). Phylogeographic agreement between prophage and bacterial housekeeping genes in *Helicobacter pylori* strains from The Gambia. Helicobacter.

[22] Vale F.F., Nunes A., Oleastro M., Gomes J.P., Sampaio D.A., Rocha R., Vítor J.M.B., Engstrand L., Pascoe B., Berthenet E. (2017). Genomic structure and insertion sites of *Helicobacter pylori* prophages from various geographical origins. Sci. Rep..

[23] Fan X., Li Y., He R., Li Q., He W. (2016). Comparative analysis of prophage-like elements in *Helicobacter* sp. genomes. PeerJ.

[24] Thiberge J., Boursaux-eude C., Lehours P., Dillies M., Creno S., Coppée Y., Rouy Z., Lajus A., Ma L., Burucoa C. (2010). From array-based hybridization of *Helicobacter pylori* isolates to the complete genome sequence of an isolate associated with MALT lymphoma. BMC Genomic.

[25] Lu W., Wise M.J., Tay C.Y., Windsor H.M., Marshall B.J., Peacock C., Perkins T. (2014). Comparative analysis of the full genome of *Helicobacter pylori* isolate Sahul64 identifies genes of high divergence. J. Bacteriol..

[26] You Y., He L., Zhang M., Zhang J. (2015). Comparative Genomics of a *Helicobacter pylori* Isolate from a Chinese Yunnan Naxi Ethnic Aborigine Suggests High Genetic Divergence and Phage Insertion. PLoS ONE.

[27] Uchiyama J., Takemura-Uchiyama I., Kato S., Takeuchi H., Sakaguchi Y., Ujihara T., Daibata M., Shimakura H., Okamoto N., Sakaguchi M. (2016). Screening of KHP30-like prophages among Japanese *Helicobacter pylori* strains, and genetic analysis of a defective KHP30-like prophage sequence integrated in the genome of the *H. pylori* strain NY40. FEMS Microbiol. Lett..

[28] Von Heinegg E.H., Nalik H.P., Schmid E.N. (1993). Characterisation of a *Helicobacter pylori* phage (HP1). J. Med. Microbiol..

[29] Luo C.-H., Chiou P.-Y., Yang C.-Y., Lin N.-T. (2012). Genome, integration, and transduction of a novel temperate phage of *Helicobacter pylori*. J. Virol..

[30] Uchiyama J., Takeuchi H., Kato S.I., Gamoh K., Takemura-Uchiyama I., Ujihara T., Daibata M., Matsuzaki S. (2013). Characterization of *Helicobacter pylori* bacteriophage KHP30. Appl. Environ. Microbiol..

[31] Abdel-Haliem M.E., Askora A. (2013). Isolation and characterization of bacteriophages of *Helicobacter pylori* isolted from Egypt. Future Virol..

[32] Cuomo P., Papaianni M., Fulgione A., Guerra F., Capparelli R., Medaglia C. (2020). An innovative approach to control *H. pylori*-induced persistent inflammation and colonization. Microorganisms.

[33] King A. (2011). Virus taxonomy: Ninth report of the International Committee on Taxonomy of Viruses.

[34] Casjens S.R., Gilcrease E.B. (2009). Determining DNA Packaging Strategy by Analysis of the Termini of the Chromosomes in Tailed-Bacteriophage Virions. Methods Mol. Biol..

[35] Minekus M., Alminger M., Alvito P., Ballance S., Bohn T., Bourlieu C., Carrière F., Boutrou R., Corredig M., Dupont D. (2014). A standardised static in vitro digestion method suitable for food-an international consensus. Food Funct..

[36] Rogers K., Levy M.I., Luebering J.E., Barton M., Bosco S., Braucher L.S., Charboneau Y., Nakamura K., Rogers K. (2011). The Digestive System.

[37] Uchiyama J., Takeuchi H., Kato S.-I., Takemura-Uchiyama I., Ujihara T., Daibata M., Matsuzaki S. (2012). Complete Genome Sequences of Two *Helicobacter pylori* Bacteriophages Isolated from Japanese Patients. J. Virol..

[38] Alves de Matos A.P., Lehours P., Timóteo A., Roxo-Rosa M., Vale F.F. (2013). Comparison of induction of B45 *Helicobacter pylori* prophage by acid and UV radiation. Microsc. Microanal..

[39] Abedon S.T., Duffy S., Turner P.E., Schaechter M. (2009). Bacteriophage Ecology. Encyclopedia of Microbiology.

[40] Kȩsik-Szeloch A., Drulis-Kawa Z., Weber-Da̧browska B., Kassner J., Majkowska-Skrobek G., Augustyniak D., Łusiak-Szelachowska M., Zaczek M., Górski A., Kropinski A.M. (2013). Characterising the biology of novel lytic bacteriophages infecting multidrug resistant Klebsiella pneumoniae. Virol. J..

[41] Hyman P., Abedon S.T. (2010). Bacteriophage Host Range and Bacterial Resistance. Adv. Appl. Microbiol..

[42] Díaz-Muñoz S.L., Koskella B. (2014). Bacteria–Phage Interactions in Natural Environments. Adv. Appl. Microbiol..

[43] Labrie S.J., Samson J.E., Moineau S. (2010). Bacteriophage resistance mechanisms. Nat. Rev. Microbiol..

[44] Lee A., O’Rourke J., De Ungria M.C., Robertson B., Daskalopoulos G., Dixon M.F. (1997). A standardized mouse model of *Helicobacter pylori* infection: Introducing the Sydney strain. Gastroenterology.

[45] Thompson L.J., Danon S.J., Wilson J.E., O’Rourke J.L., Salama N.R., Falkow S., Mitchell H., Lee A. (2004). Chronic *Helicobacter pylori* Infection with Sydney Strain 1 and a Newly Identified Mouse-Adapted Strain (Sydney Strain 2000) in C57BL/6 and BALB/c Mice. Infect. Immun..

[46] Maqbool A., Horler R.S.P., Muller A., Wilkinson A.J., Wilson K.S., Thomas G.H. (2015). The substrate-binding protein in bacterial ABC transporters: Dissecting roles in the evolution of substrate specificity. Biochem. Soc. Trans..

[47] Chan B.K., Abedon S.T., Loc-Carrillo C. (2013). Phage cocktails and the future of phage therapy. Future Microbiol..

[48] Yang H.J., Zhang J.Y., Wei C., Yang L.Y., Zuo Q.F., Zhuang Y., Feng Y.J., Srinivas S., Zeng H., Zou Q.M. (2016). Immunisation With Immunodominant Linear B Cell Epitopes Vaccine of Manganese Transport Protein C Confers Protection against *Staphylococcus aureus* Infection. PLoS ONE.

[49] Brown J.S., Ogunniyi A.D., Woodrow M.C., Holden D.W., Paton J.C. (2001). Immunization with components of two iron uptake ABC transporters protects mice against systemic *Streptococcus pneumoniae* infection. Infect. Immun..

[50] Jończyk E., Kłak M., Międzybrodzki R., Górski A. (2011). The influence of external factors on bacteriophages—review. Folia Microbiol..

[51] Nobrega F.L., Costa A.R., Santos J.F., Siliakus M.F., Van Lent J.W.M., Kengen S.W.M., Azeredo J., Kluskens L.D. (2016). Genetically manipulated phages with improved pH resistance for oral administration in veterinary medicine. Sci. Rep..

[52] Loh B., Singh Gondil V., Manohar P., Mehmood Khan F., Leptihn S. (2020). Encapsulation and Delivery of Therapeutic Phages 1 Downloaded from. Appl. Environ. Microbiol..

[53] Costa P., Pereira C., Gomes A.T.P.C., Almeida A. (2019). Efficiency of Single Phage Suspensions and Phage Cocktail in the Inactivation of *Escherichia coli* and *Salmonella Typhimurium*: An in vitro Preliminary Study. Microorganisms.

[54] Melo L., Sillankorva S., Ackermann H.-W., Kropinski A.M., Azeredo J., Cerca N. (2014). Characterization of *Staphylococcus epidermidis* phage vB_SepS_SEP9—A unique member of the Siphoviridae family. Res. Microbiol..

[55] Melo L., Sillankorva S., Ackermann H.-W., Kropinski A.M., Azeredo J., Cerca N. (2014). Isolation and characterization of a new *Staphylococcus epidermidis* broad-spectrum bacteriophage. J. Gen. Virol..

[56] Aziz R.K., Bartels D., Best A.A., DeJongh M., Disz T., Edwards R.A., Formsma K., Gerdes S., Glass E.M., Kubal M. (2008). The RAST Server: Rapid Annotations using Subsystems Technology. BMC Genom..

[57] Schattner P., Brooks A.N., Lowe T.M. (2005). The tRNAscan-SE, snoscan and snoGPS web servers for the detection of tRNAs and snoRNAs. Nucleic Acids Res..

[58] Altschul S.F., Gish W., Miller W., Myers E.W., Lipman D.J. (1990). Basic local alignment search tool. J. Mol. Biol..

[59] Soding J., Biegert A., Lupas A.N. (2005). The HHpred interactive server for protein homology detection and structure prediction. Nucleic Acids Res..

[60] Käll L., Sonnhammer E.L.L. (2002). Reliability of transmembrane predictions in whole-genome data. FEBS Lett..

[61] Kall L., Krogh A., Sonnhammer E.L.L. (2007). Advantages of combined transmembrane topology and signal peptide prediction—The Phobius web server. Nucleic Acids Res..

[62] Tusnady G.E., Simon I. (2001). The HMMTOP transmembrane topology prediction server. Bioinformatics.

[63] Petersen T.N., Brunak S., von Heijne G., Nielsen H. (2011). SignalP 4.0: Discriminating signal peptides from transmembrane regions. Nat. Methods.

[64] Klucar L., Stano M., Hajduk M. (2010). phiSITE: Database of gene regulation in bacteriophages. Nucleic Acids Res..

[65] Knudsen S. (1999). Promoter 2.0: For the recognition of PolII promoter sequences. Bioinformatics.

[66] Zuker M. (2003). Mfold web server for nucleic acid folding and hybridization prediction. Nucleic Acids Res..

[67] Naville M., Ghuillot-Gaudeffroy A., Marchais A., Gautheret D. (2011). ARNold: A web tool for the prediction of Rho-independent transcription terminators. RNA Biol..

[68] Wang Y., Coleman-Derr D., Chen G., Gu Y. (2015). OrthoVenn: A web server for genome wide comparison and annotation of orthologous clusters across multiple species. Nucleic Acids Res..

[69] McArthur A.G., Waglechner N., Nizam F., Yan A., Azad M.A., Baylay A.J., Bhullar K., Canova M.J., De Pascale G., Ejim L. (2013). The Comprehensive Antibiotic Resistance Database. Antimicrob. Agents Chemother..

[70] Xie Y., Wei Y., Shen Y., Li X., Zhou H., Tai C., Deng Z., Ou H.-Y. (2018). TADB 2.0: An updated database of bacterial type II toxin–antitoxin loci. Nucleic Acids Res..

[71] Garneau J.R., Depardieu F., Fortier L.C., Bikard D., Monot M. (2017). PhageTerm: A tool for fast and accurate determination of phage termini and packaging mechanism using next-generation sequencing data. Sci. Rep..

[72] Wagemans J., Tsonos J., Holtappels D., Fortuna K., Hernalsteens J.P., De Greve H., Estrozi L.F., Bacia-Verloop M., Moriscot C., Noben J.P. (2020). Structural Analysis of Jumbo Coliphage phAPEC6. Int. J. Mol. Sci..

[73] Shevchenko A., Tomas H., Havliš J., Olsen J.V., Mann M. (2007). In-gel digestion for mass spectrometric characterization of proteins and proteomes. Nat. Protoc..

[74] Chiva C., Olivella R., Borràs E., Espadas G., Pastor O., Solé A., Sabidó E. (2018). QCloud: A cloud-based quality control system for mass spectrometry-based proteomics laboratories. PLoS ONE.

[75] Oliveira H., Pinto G., Oliveira A., Oliveira C., Faustino M.A., Briers Y., Domingues L., Azeredo J. (2016). Characterization and genome sequencing of a *Citrobacter freundii* phage CfP1 harboring a lysin active against multidrug-resistant isolates. Appl. Microbiol. Biotechnol..

