# Supplementary material for: Characterization and Genomic Analysis of a New Phage Infecting Helicobacter pylori
Source: Int J Mol Sci. 2022 Jul 17;23(14):7885. doi: 10.3390/ijms23147885 (PMC9319048; doi:10.3390/ijms23147885)
Supplement: Supplementary file 1 [file ijms-23-07885-s001.zip › ijms-1804869-supplementary.pdf]

Table S1. Genomic features HPy1R phage.

ORF	Start (bp)	End (bp)	Size (aa)	MW (kDa)	pI	Product	Blastp	E-value (% identify)	Signals/Motifs
1	256	588	110	12.33	11	Hypothetical protein	hypothetical protein [Helicobacter phage Pt4481G]	2.00×10^{-68} (94.55%)	-
2	591	722	42	4.85	10.83	Hypothetical protein	hypothetical protein [Helicobacter phage Pt1293U]	5.00×10^{-21} (92.86%)	1 TMD
3	791	1897	392	46.04	10.55	Integrase	integrase [Helicobacter phage Pt22899G]	0 (93.16%)	-
4	1924	2079	51	6.07	10.4	Hypothetical protein	hypothetical protein [Helicobacter phage Pt1918U]	2.00×10^{-26} (92.00%)	-
5	2081	2389	102	11.84	9.95	Transcriptional regulator	hypothetical protein [Helicobacter phage Pt5771G]	1.00×10^{-53} (86.27%)	-
6	2386	3345	319	37.27	8.92	Helix-turn-helix domain	helix-turn-helix domain-containing protein [Helicobacter phage phiHP33]	0 (79.94%)	-
7	3355	3711	118	14.17	11.16	Hypothetical protein	putative ABC binding cassette transporter [Helicobacter phage Pt21299RU]	2.00×10^{-73} (91.53%)	-
8	3683	3808	41	4.98	10.4	DNA helicase	DNA helicase [Helicobacter phage Pt1846U]	2.00×10^{-19} (95.12%)	1 TMD
9	3818	4987	389	45.54	6.8	Replicative DNA helicase atpase	replicative DNA helicase [Helicobacter phage COL 5-PUJ]	0 (94.34%)	-
10	4984	6555	523	60.97	9.14	DNA primase	DNA primase [Helicobacter phage Pt21299RU]	0 (93.68%)	-
11	6567	8186	539	62.32	9.69	Exonuclease	exonuclease [Helicobacter phage Pt4497U]	0 (86.09%)	-
12	8253	13526	1757	199.59	8.8	Ddrb-parb domain-containing protein	putative crystallin beta/gamma motif-containing protein [Helicobacter phage Pt21299RU]	0 (92.49%)	-
13	13592	14869	425	48.14	4.93	Tail fiber protein	tail fiber protein [Helicobacter phage SwA626G]	0 (78.64%)	-

14	14873	15427	184	20.83	6.28	Tail fiber protein	putative tail fiber protein [Helicobacter phage PtB92G]	7.00×10^{-107} (80.87%)	-
15	15424	15819	131	15.04	9.12	Tail assembly protein	putative tail assembly protein [Helicobacter phage SwA626G]	6.00×10^{-88} (93.89%)	1 TMD
16	15816	16220	134	15.76	9.26	Hypothetical protein	hypothetical protein [Helicobacter phage DeM53M]	1.00×10^{-82} (88.81%)	-
17	16217	16804	195	22.39	4.75	Histidine kinase	putative histidine kinase [Helicobacter phage Pt21299RU]	7.00×10^{-128} (94.36%)	-
18	17045	17599	184	21.59	4.41	Structural protein	hypothetical protein [Helicobacter phage Pt1293U]	1.00×10^{-125} (96.20%)	-
19	17611	18738	375	41.53	8.38	Major capsid protein	DUF4043 family protein [Helicobacter phage phiHP33]	0 (96.00%)	-
20	18755	19129	124	13.42	7.5	Structural protein	hypothetical protein phiHP33_gp18 [Helicobacter phage phiHP33]	2.00×10^{-72} (87.9 %)	-
21	19389	19832	147	16.42	9.98	Structural protein	hypothetical protein [Helicobacter phage Pt5771G]	2.00×10^{-81} (95.24%)	1 TMD
22	19898	21709	603	69.52	5.07	Portal protein	portal protein [Helicobacter phage Pt22899G]	0 (95.69%)	-
23	21824	23380	518	60.64	8.43	Terminase	putative terminase [Helicobacter phage Pt22899G]	0 (94.59 %)	-
24	23448	23651	67	8.01	10.89	Hypothetical protein	hypothetical protein [Helicobacter phage Pt22899G]	1.00×10^{-32} (90.91%)	1 TMD
25	23663	23899	78	9.12	4.89	Hypothetical protein	hypothetical protein [Helicobacter phage Pt22899G]	2.00×10^{-34} (79.49%)	1 TMD
26	23901	24224	107	12.02	8.43	Holin	putative holin [Helicobacter phage Pt22899G]	3.00×10^{-66} (88.79%)	2 TMD
27	24226	25221	331	37.87	4.89	Structural protein	hypothetical protein [Helicobacter phage Pt5322G]	0 (85.2 %)	-
28	25218	25436	72	8.39	7.85	Hypothetical protein	hypothetical protein [Helicobacter phage Pt5322G]	2.00×10^{-44} (94.44 %)	1 SP

29	25485	26030	181	20.9	7.61	Structural protein	hypothetical protein [Helicobacter phage Pt22899G]	2.00×10^{-129} (95.58%)	-
30	26032	26829	265	29.58	7.27	Structural protein	hypothetical protein [Helicobacter phage UKEN31U]	5.00×10^{-178} (93.58 %)	-
31	26829	27392	187	21.22	10.29	Hypothetical protein	hypothetical protein [Helicobacter phage Pt1293U]	2.00×10^{-124} (91.44%)	-
32	27396	27722	108	12.48	10.3	Hypothetical protein	hypothetical protein [Helicobacter phage Pt22899G]	1.00×10^{-71} (97.22%)	1 TMD
33	27750	28667	305	35.26	5.71	Mitogen-activated protein kinase 1	mitogen-activated protein kinase 1 [Helicobacter phage SwA626G]	9.00×10^{-171} (85.32%)	1 TMD
34	28683	29549	288	32.69	9.41	Structural protein	hypothetical protein [Helicobacter phage Pt21299RU]	5.00×10^{-146} (78.11%)	-
35	29549	30412	287	32.54	5.61	Structural protein	putative transcriptional regulator protein [Helicobacter phage phiHP33]	2.00×10^{-176} (94.59%)	-
36	30491	30805	104	11.8	8.9	Hypothetical protein	-	-	-

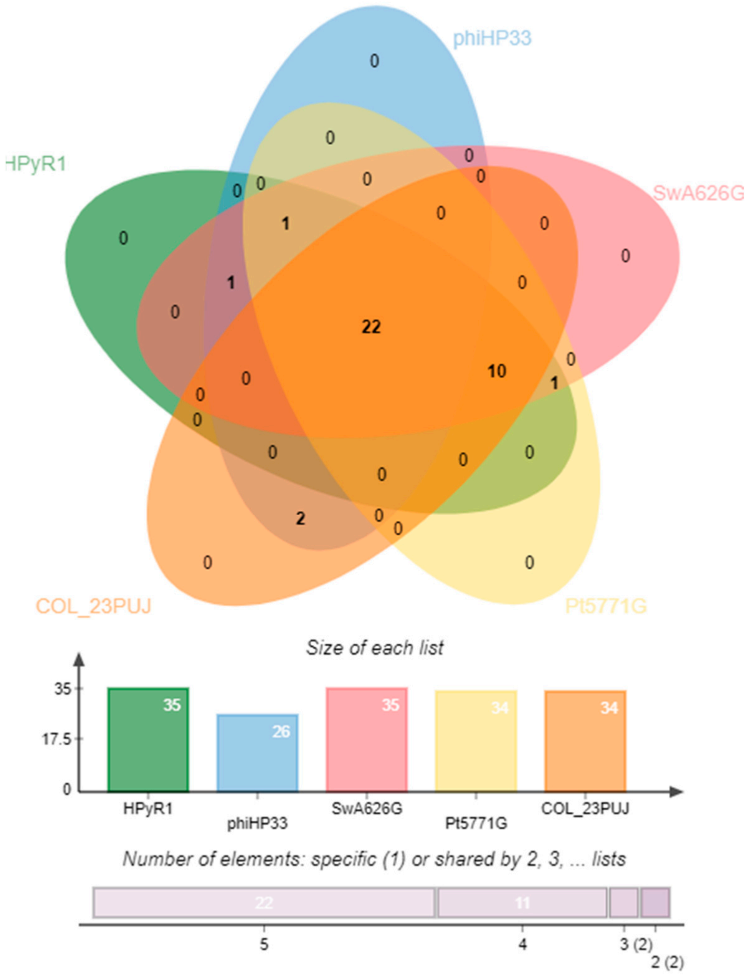

Figure S1. OrthoVenn comparison of HPy1R gene products with those of its close related phage genomes deposited at the NCBI. Venn diagram showing the number of orthologous clusters shared between the HPy1R genome and its closest related phages.
